# Video Denoising Based on a Spatiotemporal Kalman-Bilateral Mixture Model

**DOI:** 10.1155/2013/438147

**Published:** 2013-11-03

**Authors:** Chenglin Zuo, Yu Liu, Xin Tan, Wei Wang, Maojun Zhang

**Affiliations:** College of Information System and Management, National University of Defense Technology, Changsha, Hunan 410073, China

## Abstract

We propose a video denoising method based on a spatiotemporal Kalman-bilateral mixture model to reduce the noise in video sequences that are captured with low light. To take full advantage of the strong spatiotemporal correlations of neighboring frames, motion estimation is first performed on video frames consisting of previously denoised frames and the current noisy frame by using block-matching method. Then, current noisy frame is processed in temporal domain and spatial domain by using Kalman filter and bilateral filter, respectively. Finally, by weighting the denoised frames from Kalman filtering and bilateral filtering, we can obtain a satisfactory result. Experimental results show that the performance of our proposed method is competitive when compared with state-of-the-art video denoising algorithms based on both peak signal-to-noise-ratio and structural similarity evaluations.

## 1. Introduction

Recently, as the rapid development of digital imaging technology, digital imaging devices have been widely applied in many fields, including computational photography, security monitoring, robot navigation, and military reconnaissance. However, video signals are often contaminated by all kinds of noise during acquisition and transmission, such as optical noise, component noise, sensor noise, and circuit noise. The noise in video signals not only damages the original information and results in unpleasant visual effect, but also affects the effectiveness of further coding or processing such as feature extraction, object detection, motion tracking, and pattern recognition. So, noise reduction in contaminated video sequences should be implemented.

Many video denoising methods have been proposed in the past decade, most of which perform in the spatial domain, temporal domain, or their combination [1–6]. Methods in spatial domain often produce limited results because they do not take advantage of spatiotemporal correlations of neighboring frames. Methods in temporal domain consider the correlations of neighboring frames, but they are only appropriate for still video. Additionally, the results have artifacts or smear phenomenon when objects motion exist. By combining the spatial domain with temporal domain, impressive results can be produced. However, these methods generally require a huge amount of computation. With the emergence of new multiresolution tools, such as the wavelet transform [7, 8], video denoising methods performing in transform domain were proposed continually [9–12]. Now, the transform domain techniques in general, especially the wavelet-based video denoising methods, have been shown to outperform these spatiotemporal video denoising methods. Moreover, methods that combine spatiotemporal domain and transform domain were also proposed [13–16], which could produce perfect denoising effect. Similarly, this kind of methods also require huge amount of computation.

However, although video denoising technology has made great progress, most of these methods are unable to obtain ideal effect for large noisy video sequences in low light, which is urgently needed in many fields, especially in the security monitoring field. In this field, the monitoring devices are fixed in some places in general, so the captured video sequences have fixed background. In practical applications, it often requires to see the characteristic both of still and moving objects in the video sequences clearly. This requirement can be met easily in the day time. However, in the night time, because of the low light condition, captured video sequences are contaminated by noise badly. To some extent, existing video denoising methods can reduce the noise of contaminated video sequences, but this is far from enough to meet the requirement.

In this paper, a novel video denoising method based on a spatiotemporal Kalman-bilateral mixture model is proposed. Firstly, we perform an appropriate average filtering on current noisy frame to reduce the influence of noise, which we call prefiltering. This step is useless to the final denoising result, but preparative to the motion estimation. Then, take advantage of the strong spatiotemporal correlations of neighboring frames, block-matching based motion estimation is performed by comparing current pre-filtered frame with previously denoised frames. Based on motion estimation results, current noisy frame is processed in the temporal domain by using Kalman filter [17] on the one hand. It is noteworthy that different blocks of the noisy frame have different filtering strength according to their block-matching results. In the Kalman filtering, motion blocks have quite weak filtering strength to keep their motion characteristic, while still blocks have strong filtering strength to reduce the noise. On the other hand, current noisy frame is also processed in the spatial domain by using bilateral filter [18], which aims at reducing the noise globally. Finally, by weighting the two denoised frames from Kalman filtering and bilateral filtering, we can obtain a satisfactory result, in which the still region is largely from Kalman filtered result and the motion region is almost from bilateral filtered result. Experimental results show that the performance of our proposed method is effective over current competing video denoising methods.

The remainder of the paper is organized as follows.  Section 2 reviews related work.  Section 3 describes our proposed spatiotemporal Kalman-bilateral mixture model.  Section 4 provides quantitative quality evaluations of the denoising results. In Section 5, experiments are implemented and the experimental results are shown. Finally, Section 6 concludes this article.

## 2. Related Work

Buades et al. [2] firstly proposed the Non Local Means (NLM) method. This method replaced a noisy pixel by the weighted average of pixels with related surrounding neighborhoods, and finally could produce quite satisfactory denoising results. However, high computational complexity makes this method impractical. Later, Karnati et al. [3] improved the NLM algorithm. They replaced the window similarity by a modified multiresolution based approach with much fewer comparisons rather than all pixels comparisons. In their method, mean values of the variable sized windows were computed efficiently using summed image (SI) concept, which requires only 3 additions. Finally, the computational speed was increased by 80 times. Based on the NLM algorithm, many methods were proposed for video denoising [4–6, 13]. Mahmoudi and Sapiro [4] introduced filters that eliminated unrelated neighborhoods from the weighted average to accelerate the original NLM algorithm and applied it for video denoising. Yin et al. [5] proposed a novel scheme by using the mean absolute difference (MAD) of the current pixel block and the candidate blocks both in spatial and temporal domain as a preselecting criterion. Rather than one single pixel, this scheme reconstructed a block with different number of pixels according to the statistic property of the current pixel block, which dramatically lowered the computational burden and kept good denoising performance. Dabov et al. [13] proposed an effective video denoising method based on highly sparse signal representation in local 3D transform domain. They developed a two-step video denoising algorithm where the predictive search block-matching was combined with collaborative hard-thresholding in the first step and with collaborative wiener filtering in the second step. Finally, state-of-the-art denoising results were achieved. Moreover, Guo et al. [19] proposed a recursive temporal denoising filter named multihypothesis motion compensated filter (MHMCF). This filter fully exploited temporal correlation and utilized a number of reference frames to estimate the current pixel. As a purely temporal filter, it well preserved spatial details and achieved satisfactory visual quality.

In addition, there are still many video denoising methods performing in transform domain [9–12, 14–16]. Zlokolica et al. [9] introduced a new wavelet based motion reliability measures and performed motion estimation and adaptive recursive temporal filtering in a closed loop, followed by an intra-frame spatially adaptive filter. Mahbubur Rahman et al. [10] proposed a joint probability density function to model the video wavelet coefficients of any two neighboring frames and then applied this statistical model for denoising. Jovanov et al. [11] reused motion estimation resources from the video coding module for video denoising. They proposed a novel motion field filtering step and a novel recursive temporal filter with appropriately defined reliability of the estimated motion field. Luisier et al. [12] proposed an efficient orthonormal wavelet-domain video denoising algorithm. This method took full advantage of the strong spatiotemporal correlations of neighboring frames and could outperform most state-of-the-art wavelet-based techniques. Yu et al. [14] integrated both the spatial filtering and recursive temporal filtering into the 3-D wavelet domain and effectively exploited both the spatial and temporal redundancies. Varghese and Wang [15] applied motion estimation to enhance the correlations between temporal neighboring wavelet coefficients and proposed a spatiotemporal Gaussian scale mixture model for natural video signals. Maggioni et al. [16] separately exploited the temporal and nonlocal correlation of the video and constructed 3-D spatiotemporal volumes by tracking blocks along trajectories defined by the motion vectors. In addition, other video denoising methods, such as the method by using low-rank matrix completion [20], were also proposed recently and achieved good results.

However, most existing video denoising methods cannot achieve satisfactory results when the video sequences are contaminated badly in low light. In this paper, we propose a spatiotemporal Kalman-bilateral mixture model, which can reduce the noise in large noisy video sequences that are captured with low light.

## 3. Proposed Spatiotemporal Kalman-Bilateral Mixture Model

**Figure 1:**
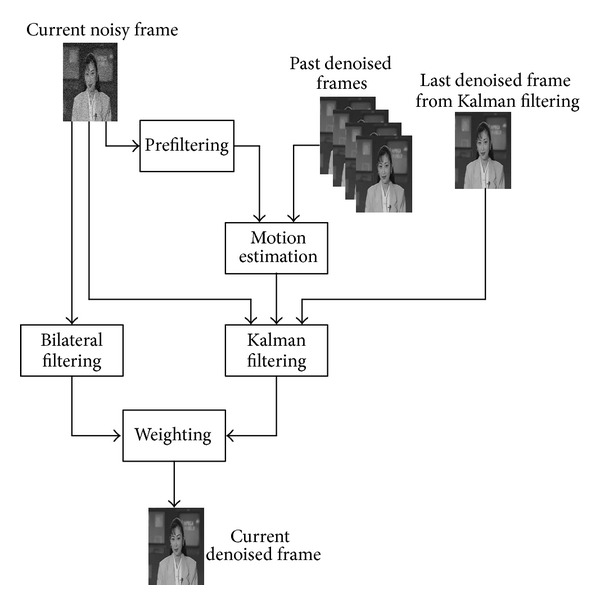
Diagram of proposed ST-KBM video denoising algorithm.

Figure 1 illustrates the diagram of our proposed spatiotemporal Kalman-bilateral mixture (ST-KBM) model. The denoising of current noisy frame involves not only the frame itself, but also a series of past denoised frames. Firstly, prefiltering is performed on current noisy frame. The purpose of this operation is to reduce the influence of noise as possible and prepare for next motion estimation. Motion estimation is performed between the current noisy frame and past denoised frames, and the estimation results are used to guide the Kalman filtering on current noisy frame. In addition, bilateral filtering is also performed on current noisy frame. So, after above processing, there are two denoised frames, one comes from Kalman filtering and another comes from bilateral filtering. Finally, by weighting the two denoised frames, we can obtain a satisfactory result.

### 3.1. Motion Estimation

Motion estimation itself is a complex problem. Generally, motion estimation is performed directly. When the video has relatively little noise, estimation results will be accurate. However, as the increase of noise, the precision of motion estimation becomes quite low. With the influence of large noise, precision motion estimation is becoming difficult. So, we perform average filtering on the current noisy frame to restrain the influence of noise as possible before motion estimation, which is called prefiltering. After the prefiltering step, the large noise is restrained by a huge margin while the motion in the video remains well. In this case, although the frame has become quite fuzzy, motion estimation is not affected. Note that the prefiltering procedure is only implemented for motion estimation, rather than contributing for the image-signal denoising.

Then, take advantage of the strong correlations between adjacent frames, motion estimation based on block-matching is performed by comparing current pre-filtered frame with past denoised frames. Block-matching (BM) [21] is a particular matching approach that has been extensively used for motion estimation in video compression. Here, we use it to calculate whether motion exists in the block.

**Figure 2:**
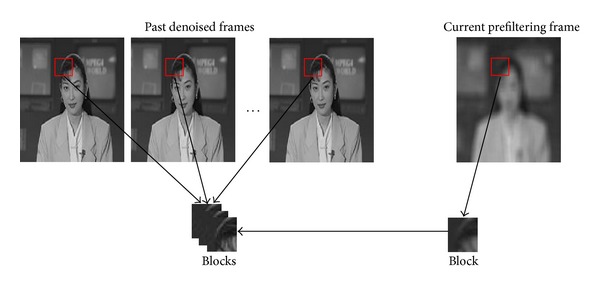
Block-matching for motion estimation by comparing current prefiltered frame with past denoised frames.

An illustrative example of block-matching is given in Figure 2. Firstly, divide current pre-filtered frame and past denoised frames into a number of blocks which have fixed size *N* × *N*. Then, we compare the block in current prefiltering frame with blocks that have the same position in past denoised frames, respectively, and use *ℓ* ^2^-distance as the measure whether motion exists in the block, which is called motion measure. The block distance can be calculated as

(1) $$\begin{array}{c} d\left(B_{\text{current}}^{m},B_{\text{past},i}^{m}\right)=\frac{{||v\left(B_{\text{current}}^{m}\right)-v\left(B_{\text{past},i}^{m}\right)||}_{2}^{2}}{N^{2}}, \end{array}$$

where ||·||_2_ denotes the *ℓ* ^2^-norm, *v*(*B*_current_^*m*^) and *v*(*B*_past,*i*_^*m*^) are the intensity gray level vectors of the *m*th block in current prefiltering frame and that in the *i*th past denoised frame, respectively. After calculating the block distances between current prefiltering frame and each past denoised frame, respectively, final motion measure of the *m*th block in current prefiltering frame can be gain by averaging them as follows:

(2) $$\begin{array}{c} d_{m}=\frac{\sum_{i=1}^{n}d\left(B_{\text{current}}^{m},B_{\text{past},i}^{m}\right)}{n}. \end{array}$$

The averaged block distance measure the extent that motion exists in the block of current prefiltering frame. The larger the value is, the greater the likelihood is. Therefore, by calculating all of the block distances in current prefiltering frame, we can get global motion estimation.

### 3.2. Motion Estimation Based Kalman Filtering in Temporal Domain

The discrete Kalman filter [17] is a set of mathematical equations that provides an efficient computational solution of the least squares method. It can estimate the state of a dynamic system from a series of incomplete noisy measurements by using a form of feedback control. This procedure consists of two consecutive stages: prediction and updating. The prediction stage projects forward the current state and error covariance estimates to obtain a priori estimate for the next time step in time. The updating stage incorporates a new measurement into the priori estimate to obtain an improved posteriori estimate.

 The prediction equations can be presented as follows:

(3) $$\begin{array}{c} x_{k}^{-}=A_{k}\cdot x_{k-1}^{+}+B_{k}\cdot u_{k},\\ p_{k}^{-}=A_{k}\cdot p_{k-1}^{+}A_{k}^{T}+Q_{k}. \end{array}$$

In the above equations, the superscripts “−” and “+” in the equations denote “before” and “after” each measurement, respectively. *x*_*k*−1_^+^ and *p*_*k*−1_^+^ represent the estimated state matrix and state covariance matrix of last state, respectively. *x*_*k*_^−^ and *p*_*k*_^−^ represent the priori estimates of state matrix and state covariance matrix for current state. *A*_*k*_ represents the state transition matrix which determines the relationship between the present state and the previous one. Matrix *B*_*k*_ relates the control input *u*_*k*_ to current state. *Q*_*k*−1_ represents the covariance matrix of process noise.

In our case, we try to estimate current video frame based on the last one. So, the state matrix in above equations is just the video frame matrix. In the video sequences, there is not any control input, which means *u*_*k*_ = 0. For the priori estimates for current state, we assume it is the same as last state. So, we can obtain following equations:

(4) $$\begin{array}{c} x_{k}^{-}=x_{k-1}^{+},\\ p_{k}^{-}=p_{k-1}^{+}+Q_{k}. \end{array}$$

The process noise in the video sequences is just resulted by the motion. So, for any pixel (*x*, *y*) in the *m*th block of current noisy frame, we define

(5) $$\begin{array}{c} Q_{k-1}\left(x,y\right)=d_{m}, \end{array}$$

which keeps the covariance of motion region larger than that of still region. 

The updating equations can be presented as follows:

(6) $$\begin{array}{c} \text{K}{\text{g}}_{k}=p_{k}^{-}H_{k}^{T}{\left(H_{k}p_{k}^{-}H_{k}^{T}+R_{k}\right)}^{-1},\\ x_{k}^{+}=x_{k}^{-}+\text{K}{\text{g}}_{k}\left(z_{k}-H_{k}x_{k}^{-}\right),\\ p_{k}^{+}=\left(I-\text{K}{\text{g}}_{k}H_{k}\right)p_{k}^{-}. \end{array}$$

The first task during the updating stage is to compute the Kalman gain, Kg_*k*_, which is known as the blending factor to minimize the posteriori error covariance. In the above equations, *x*_*k*_^−^ and *p*_*k*_^−^ are the priori estimates calculated in prediction stage. Matrix *H*_*k*_ describes the relationship between the measurement vector, *z*_*k*_, and the posteriori state vector, *x*_*k*_^+^. *R*_*k*_ is the covariance matrix of measurement noise. *p*_*k*_^+^ is the posteriori estimates of state covariance matrix for current state.

In our case, *z*_*k*_ and *x*_*k*_^+^ represent current noisy and denoised frames, respectively. *H*_*k*_ is the unit matrix. The measurement noise just represents the noise in the video sequences. So, we can obtain the following equations:

(7) $$\begin{array}{c} \text{K}{\text{g}}_{k}=p_{k}^{-}{\left(p_{k}^{-}+R_{k}\right)}^{-1},\\ x_{k}^{+}=x_{k}^{-}+\text{K}{\text{g}}_{k}\left(z_{k}-x_{k}^{-}\right),\\ p_{k}^{+}=\left(I-\text{K}{\text{g}}_{k}\right)p_{k}^{-}. \end{array}$$

After Kalman filtering, we can obtain a denoised frame, in which the still region is denoised quite well. However, the moving region still has much noise because Kalman filter retains the information of this region intact. Therefore, for the motion region, we use the bilateral filter to reduce its noise as possible.

### 3.3. Bilateral Filtering in Spatial Domain

The bilateral filter was introduced by Tomasi and Manduchi [18] as a noniterative means of smoothing images while retaining edge detail. It involves a weighted convolution in which the weight for each pixel depends not only on its distance from the center pixel, but also its relative intensity. So, for any pixel (*x*, *y*) in the frame, its filtered intensity value *V*(*x*, *y*) can be calculated as follows:

(8) $$\begin{array}{c} V\left(x,y\right)=\frac{\sum_{(i,j)\in S_{x,y}}w\left(i,j\right)\cdot V\left(i,j\right)}{\sum_{(i,j)\in S_{x,y}}w\left(i,j\right)}. \end{array}$$

In above equation, *S*_*x*,*y*_ represents the neighbourhood centered in the pixel. *V*(*i*, *j*) represents the intensity value of pixel (*i*, *j*) in the neighborhood. The weighting coefficient *w*(*i*, *j*) consists of two parts, as shown in follows:

(9) $$\begin{array}{c} w\left(i,j\right)=w_{s}\left(i,j\right)\cdot w_{r}\left(i,j\right)\\ w_{s}\left(i,j\right)=e^{-\left({\left(i-x\right)}^{2}+{\left(j-y\right)}^{2}\right)/2\sigma_{s}^{2}}\\ w_{r}\left(i,j\right)=e^{-{\left[V(i,j)-V(x,y)\right]}^{2}/2\sigma_{r}^{2}}, \end{array}$$

*w*_*s*_(*i*, *j*) is the weighting coefficient depended on the distance difference from the center pixel, while *w*_*r*_(*i*, *j*) is the weighting coefficient depended on the intensity different from the center pixel. *σ*_*s*_ and *σ*_*r*_ are the variation coefficient of the two weighting coefficient, which control their degree of attenuation.

Only reducing the noise in the moving region of denoised frame from Kalman filtering is complicated. So, we apply the bilateral filter on whole current noisy frame. In this case, both the still region and moving region are denoised. Then, by weighting the two denoised frames from Kalman filtering and bilateral filtering, an integrated denoised frame can be obtained, in which the still region is from Kalman filtering and the moving region is from bilateral filtering.

### 3.4. Weighted Average

After Kalman filtering and bilateral filtering, we have two denoised frames. One is from Kalman filtering, in which the still regions are well denoised but the motion regions remain the noisy information intactly. Another is from bilateral filtering, in which the motion regions are denoised to some extent. So, we integrate the two denoised frames by weighting them based on motion estimation results. The weight is based on Gaussian distribution, and for any pixel (*i*, *j*) in the *m*th block, its weight value, *w*_*c*_(*i*, *j*), can be calculated as follows:

(10) $$\begin{array}{c} w_{c}\left(i,j\right)=e^{-d_{m}^{2}/\sigma_{c}^{2}}. \end{array}$$

**Figure 3:**
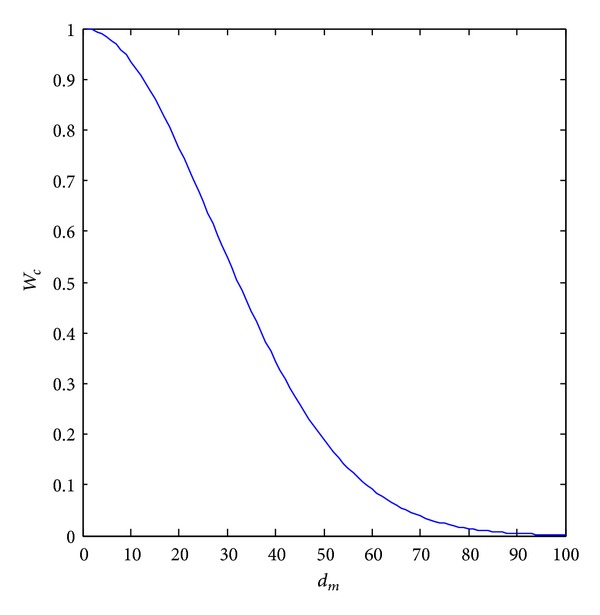
The weight calculated based on motion estimation value.

Based on the above equation, the motion and still regions can be further distinguished effectively. As shown in Figure 3, the larger the value of motion estimation is, the smaller the weight is. *σ*_*c*_ is used to control the degree of attenuation.

Then, the weighted denoised frame can be calculated as follows

(11) $$\begin{array}{c} X_{c}=W_{c}\cdot X_{\text{kalman}}+\left[I-W_{c}\right]\cdot X_{\text{bilateral}}. \end{array}$$

**Figure 4:**
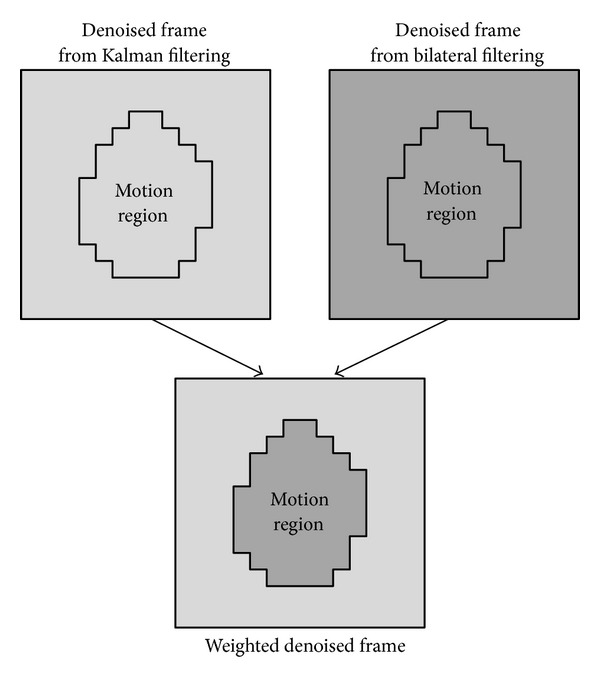
Weight the two denoised frames based on motion estimation.

Here, *W*_*c*_ represents the weight matrix calculated by (10). *X*_kalman_ and *X*_bilateral_ represents the denoised frame matrices by Kalman filtering and bilateral filtering, respectively. *X*_*c*_ is just the desired weighted frame matrix. After weighted average, both the motion region and still region of the weighted frame have been denoised, as shown in Figure 4.

## 4. Validation Criteria

For providing quantitative quality evaluations of the denoising results, two objective criteria, namely the PSNR and the SSIM [22–24], are employed. PSNR is defined as

(12) $$\begin{array}{c} \text{PSNR}=10\cdot \text{lo}{\text{g}}_{10}\left(\frac{L^{2}}{\text{MSE}}\right), \end{array}$$

where *L* is the dynamic range of the image (for 8 bits/pixel images, *L* = 255) and MSE is the mean squared error between the original and distorted images. SSIM is first calculated within local windows using

(13) $$\begin{array}{c} \text{SSIM}\left(x,y\right)=\frac{\left(2\mu_{x}\mu_{y}+C_{1}\right)\left(2\sigma_{xy}+C_{2}\right)}{\left(\mu_{x}^{2}+\mu_{y}^{2}+C_{1}\right)\left(\sigma_{x}^{2}+\sigma_{y}^{2}+C_{2}\right)}, \end{array}$$

where *x* and *y* are the image patches extracted from the local window from the original and noisy images, respectively. *μ*_*x*_, *σ*_*x*_^2^, and *σ*_*xy*_ are the mean, variance, and cross-correlation computed within the local window, respectively. The overall SSIM score of a video frame is computed as the average local SSIM scores. PSNR is the mostly widely used quality measure in the literature, but has been criticized for not correlating well with human visual perception [25]. SSIM is believed to be a better indicator of perceived image quality [25]. It also supplies a quality map that indicates the variations of images quality over space. The final PSNR and SSIM results for a denoised video sequence are computed as the frame average of the full sequence.

## 5. Experiments and Results

In order to evaluate the performance of our proposed ST-KBM algorithm, we compare it with some state-of-the-art video denoising algorithms, such as ST-GSM [15] and VBM3D [13]. The original codes of these two algorithms can be downloaded online [26, 27].

In the experiments, four video sequences are selected from the publicly available video sequences [28], which have fixed background. The noisy video sequences are simulated by adding independent white Gaussian noises of given variance *σ* ^2^ on each frame.  Table 1 shows the PSNR and SSIM results of proposed ST-KBM, ST-GSM, and VBM3D for the four video sequences at five noise levels. When the noise level is relatively low, the proposed ST-KBM algorithm works well but still has a gap with ST-GSM and VBM3D. However, when the noise level is high, it performs better than ST-GSM and VBM3D for most of the test sequences. In particular, the SSIM of ST-KBM is much better than other two algorithms.

**Figure 5:**
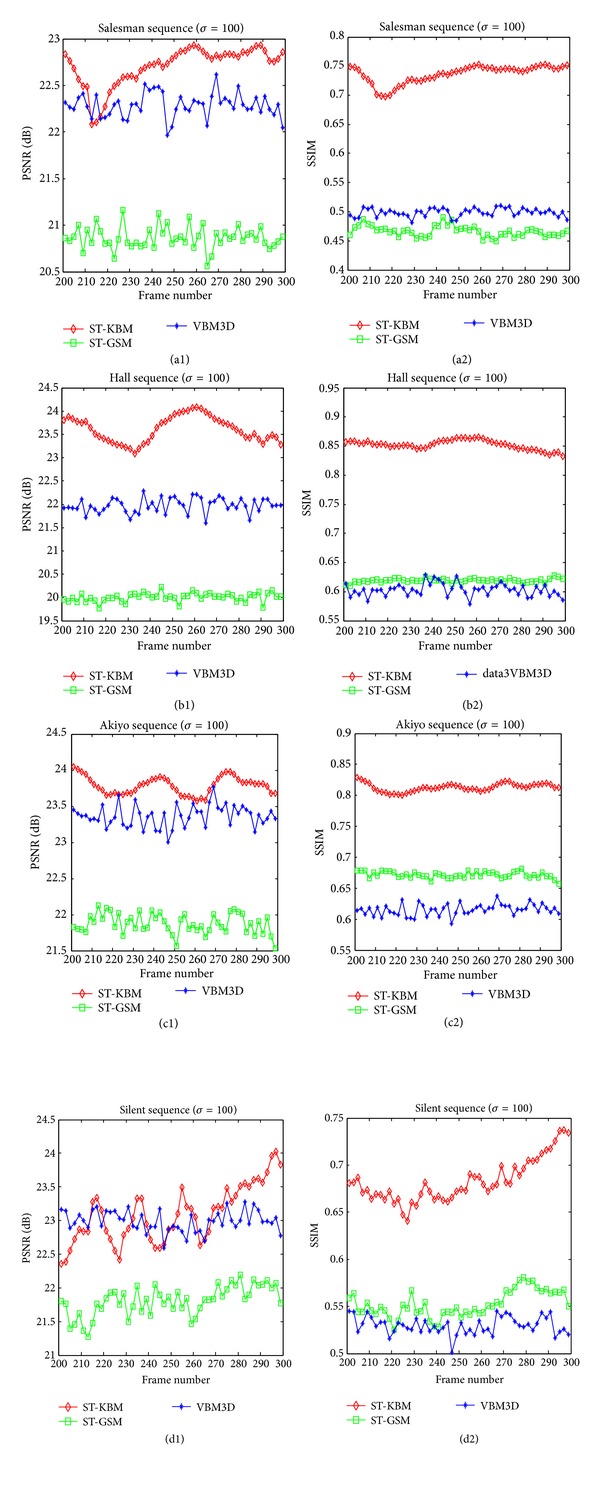
Comparison of PSNR and SSIM evolution for four video sequences corrupted with noise standard deviation *σ* = 100 and three denoising algorithms. (a1)-(a2) PSNR and SSIM of denoised Salesman sequence. (b1)-(b2) PSNR and SSIM of denoised Hall sequence. (c1)-(c2) PSNR and SSIM of denoised Akiyo sequence. (d1)-(d2) PSNR and SSIM of denoised Silent sequence.

**Figure 6:**
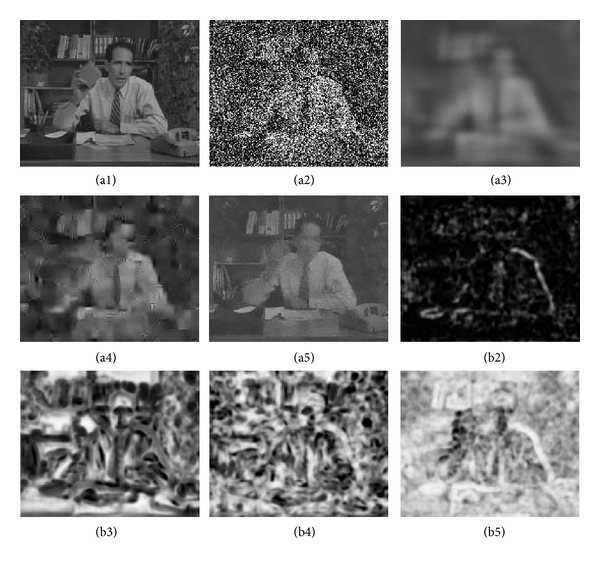
Denoising results of frame 105 in Salesman sequence corrupted with noise standard deviation *σ* = 100. (a1)–(a5) Image frames in the original, noisy, ST-GSM [15], VBM3D [13], and ST-KBM denoised sequences. (b2)–(b5) Corresponding SSIM quality maps (brighter indicates larger SSIM value).

In Figure 5, we show the PSNR and SSIM from frame 200 to 300 of the test video sequences corrupted by noise with *σ* = 100. With the comparison to PSNR, our proposed ST-KBM algorithm performs slightly better than ST-GSM and VBM3D. However, for SSIM, it outperforms ST-GSM and VBM3D obviously, which means that the denoised video sequences by using ST-KBM algorithm have better visual quality.  Figure 6 demonstrates the visual effects of the three video denoising algorithms. In particular, we show the frame 105 extracted from the Salesman sequence, together with a noisy version of the same frame, and the denoised frames obtained by the three video denoising algorithms. It can be seen that our proposed ST-KBM algorithm is obviously effective at suppressing background noise while maintaining the structural information of the scene. This is further verified by examining the SSIM quality maps of the corresponding frames. The results show that our proposed ST-KBM algorithm is perfectly effective to the large noisy video sequences and can achieve state-of-the-art denoising performance.

**Figure 7:**
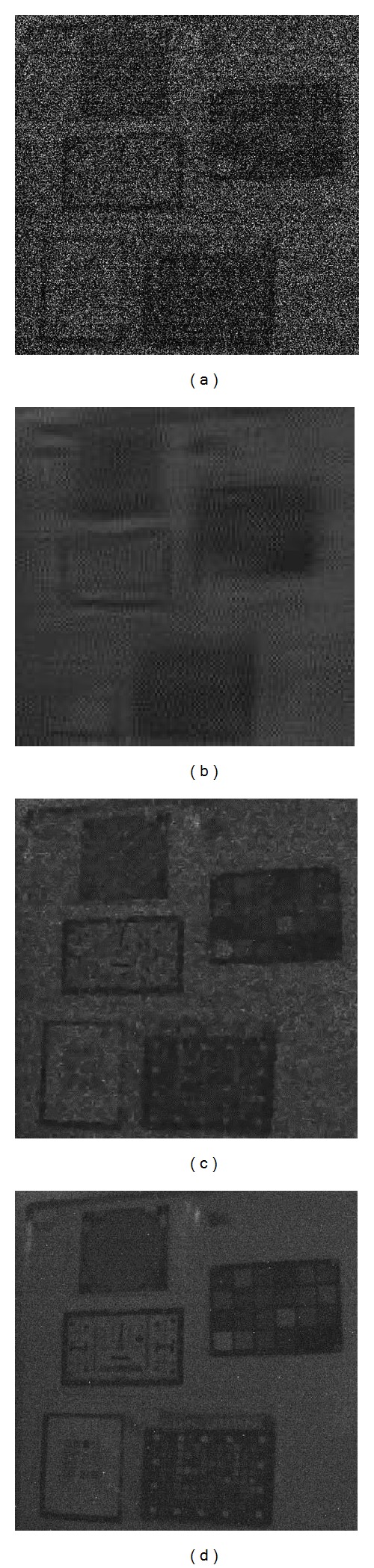
Denoising results of a natural noisy video sequence in low light. (a)–(d) Image frames in the noisy, ST-GSM [15], VBM3D [13], and ST-KBM denoised sequences.

Moreover, to further demonstrate the practicability of proposed ST-KBM algorithm, we implement practical experiments, as shown in Figure 7. The natural noisy video sequence is captured in very low light, and the real information is damaged badly. It is worth mentioning that the noise in the sequence is mixed, including white Gaussian noise, Possion noise, and other kinds of noise, which means noise reduction is more difficult. Obviously, objects in ST-KBM denoised sequence, such as the resolution charts and color charts, have clearer shape than those in ST-GSM and VBM3D denoised sequences. The denoising results show that our proposed ST-KBM algorithm is also quite effective for the mixed noise and can produce better visual effect than ST-GSM and VBM3D.

## 6. Conclution

In this paper, we have presented a ST-KBM model for large noisy video signals that have fixed background, and applied it to the restoration both of simulated noisy video sequences by additive white Gaussian noise and natural noisy video sequence in low light. Thanks to the operation of prefiltering, the motion estimation by comparing current pre-filtered frame with previously denoised frames is performed effectively. Then, Kalman filter and bilateral filter are applied for current noisy frame, respectively. Finally, by weighting the denoised frames from Kalman filtering and bilateral filtering, a satisfactory result is obtained. The experimental comparisons with state-of-the-art algorithms show that our proposed ST-KBM is competitive for large noisy video sequences that have a fixed background in terms of both subjective and objective evaluations.

## Figures and Tables

**Table 1:** PSNR and SSIM comparisons of video denoising algorithms for 4 video sequences at 5 noise levels.

Video sequence noise std (*σ*)	Salesman					Hall				
	10	15	20	50	100	10	15	20	50	100
PSNR results (dB)										
ST-GSM [15]	37.93	35.56	33.89	26.43	20.72	38.28	35.99	34.12	27.16	19.99
VBM3D [13]	**39.11**	**36.65**	**34.72**	27.93	22.18	**39.96**	**37.93**	**36.31**	28.14	21.97
ST-KBM	35.48	33.81	33.52	**29.64**	**22.73**	35.73	33.21	32.84	**28.44**	**23.42**

SSIM results										
ST-GSM [15]	0.970	0.950	0.928	0.699	0.452	0.975	0.965	0.955	0.882	0.620
VBM3D [13]	**0.976**	**0.958**	0.932	0.742	0.489	**0.980**	**0.973**	0.966	0.887	0.601
ST-KBM	0.954	0.942	**0.934**	**0.864**	**0.734**	0.978	0.970	**0.967**	**0.929**	**0.830**

Video Sequence Noise std (*σ*)	Akiyo					Silent				
	10	15	20	50	100	10	15	20	50	100

PSNR results (dB)										
ST-GSM [15]	40.67	38.34	36.53	28.44	21.89	37.41	35.17	33.61	27.63	21.87
VBM3D [13]	**42.00**	**39.72**	**37.85**	**30.69**	23.36	**38.67**	**36.34**	**34.59**	**28.38**	23.08
ST-KBM	35.09	34.66	34.06	30.17	**23.67**	34.26	32.78	32.29	27.98	**23.10**

SSIM results										
ST-GSM [15]	0.980	0.969	0.958	0.852	0.673	0.963	0.943	0.922	0.787	0.561
VBM3D [13]	**0.984**	**0.976**	**0.964**	0.871	0.614	**0.970**	**0.951**	**0.928**	0.773	0.535
ST-KBM	0.962	0.954	0.943	**0.894**	**0.795**	0.937	0.922	0.907	**0.823**	**0.692**
